# Biomimicry of *Echinocactus grusonii* Spines as a Source of Inspiration for Design Principles and Implantation Strategies of Self-Inserting Intraneural Interfaces

**DOI:** 10.3390/biomimetics10110773

**Published:** 2025-11-14

**Authors:** Pier Nicola Sergi

**Affiliations:** Translational Neural Engineering Area, The BioRobotics Institute and Department of Excellence in Robotics and Artificial Intelligence, Sant’Anna School of Advanced Studies, Piazza Martiri della Libertà, 33, 56127 Pisa, Italy; pn.sergi@gmail.com or pn.sergi@cauchyinstitute.it

**Keywords:** biomimicry, *Echinocactus grusonii*, spines, buckling, neural interfaces, peripheral nerves

## Abstract

Cactaceae are plants equipped with spines and adapted to extremely arid environments. In particular, *Echinocactus grusonii* spines are almost cylindrical structures, which may occasionally present an enlargement of their proximal cross sectional area. In this work, the spines of *Echinocactus grusonii* were explored as a possible source of biomimetic inspiration for the design and the implantation strategies of self-inserting intraneural interfaces. More specifically, the elastic stability of spines was theoretically studied for structures able to puncture the surface of an external object, as well as for structures unable to pierce it. The biomimicry of *Echinocactus grusonii* spines suggested an improved insertion strategy for self-inserting intraneural interfaces together with structural changes able to increase their elastic stability. The theoretical approach provided in this work was able to predict an increase of the first buckling threshold up to 39% for not puncturing self-inserting neural interfaces, and up to 59% for puncturing ones.

## 1. Introduction

The family of Cactaceae comprises around 1800 species of plants [1,2] characterized by stem succulence, stem-based photosynthesis, and reduction of leaves in spines [3,4]. They have unusual growth forms, reflecting their adaptation to extremely arid environments [5], together with the ability to produce various type of flowers to attract different kinds of pollinators [6,7]. These characteristics made these plants ornamental and widespread throughout the world [8], so much so that some species have become invasive [9,10]. To avoid excessive loss of water through evaporation, these plants are covered by a skin-like layer called cuticle [11], which could have a variable thickness [12], and whose mechanical toughness and underlying structural organization has been recently investigated [13]. A unique feature of these plants is the presence of areoles [14,15], which can produce new branches, flowers [16] and spines [17,18]. In general, spines are defensive organs able to protect plants against browsing by small climbing mammals [19], as well as to provide a mechanical defense against herbivores [20]. Cactaceae spines are able to avoid excessive sunlight irradiation [21], to collect moisture and condense water [3,22], to increment seed dispersal and vegetative reproduction through the rooting of detached stem segments [23]. Different species of Cactaceae have different macroscopic characteristics and spines with different size, color, and textures together with gross and micro morphology [24,25].

As a consequence, morphology and structure of Cactaceae have been extensively studied in the past [26,27,28]. Similarly, the anatomy of spines have been investigated [21,29] and their basic components (e.g., libriform fibers and sclerified epidermis) were thought to govern their penetration stability.

Spines share their ability to enter biological tissues with intraneural interfaces, which are high-tech devices designed to connect neural fibers to external devices [30,31,32,33]. Unlike regenerative interfaces [30,34,35], which exploit the ability of axons to regenerate through their conductive structures [36,37,38,39,40], intraneural interfaces aim at connecting axons through mechanical contact. In particular, their conductive structures are inserted within nerves through microneedles [41,42,43] (intraneural interfaces) or, directly, through their main shaft (self-inserting intraneural interfaces). Peripheral nerves, indeed, are extremely complex biological structures, because of their internal axonal organization [44,45,46,47], their internal system of blood perfusion [48] and the organization of their connective tissues [49]. Nevertheless, their internal axons can be reached thanks to the soft nature of the peripheral nervous tissue [50] mainly governed by elastin [51,52,53,54] and axially reinforced by collagen fibers [55,56].

The similarity between external geometry and mechanics of self-inserting intraneural interfaces and spines, results in a similar behaviour in interactions with external objects. This behaviour is related both to the nature of building materials and to the geometry of the main shaft of the self-inserting intraneural interfaces. Analogously, it is related to the building materials and to the geometry of the inserting micro-needles, in case of intraneural interfaces, unable to be self-inserted [42,43,57]. More specifically, for both spines and self-inserting intraneural interfaces, the insertion force increases together with the radius of their main shaft, while a larger shaft stiffness results in a larger buckling threshold, that is the ability to withstand axial force without any deflection. In addition, the texture of the surface is able to influence the stability of the post-penetration phase.

Recently, some improvements were obtained using biomimicry as a source of inspiration for structural design and insertion procedures of neural interfaces targeting the central nervous tissue [58]. More specifically, some innovations were inspired by the skin of the echinoderm Holothuroidea (sea cucumber), which suggested the implementation of a polymeric material with variable stiffness to change the compliance of the main shaft as a function of the biochemical compounds present within the biological environment [59]. In this way, they were stiff enough to be inserted within the central nervous tissue and progressively more compliant to minimize the stiffness mismatch with the surrounding environment. These characteristics were able to avoid the onset of the extremely complex foreign body response and the encapsulation of the neural interface [60,61,62,63,64,65,66,67,68,69,70,71,72,73,74,75,76]. Also, the mosquito behaviour was used to minimize failure and increase the ability of structures to withstand insertion force. Indeed, this insect uses an additional structure (the labium) [77,78] to increase the stiffness of the proboscides, which can easily be prone to elastic instability if inserted directly into the skin of animals [79,80]. Finally, the wasp ovipositor [81,82] was imitated to improve the design of the interface structures and surgical needles [83,84] and improve stabilization within the tissue [85].

**Figure 1:**
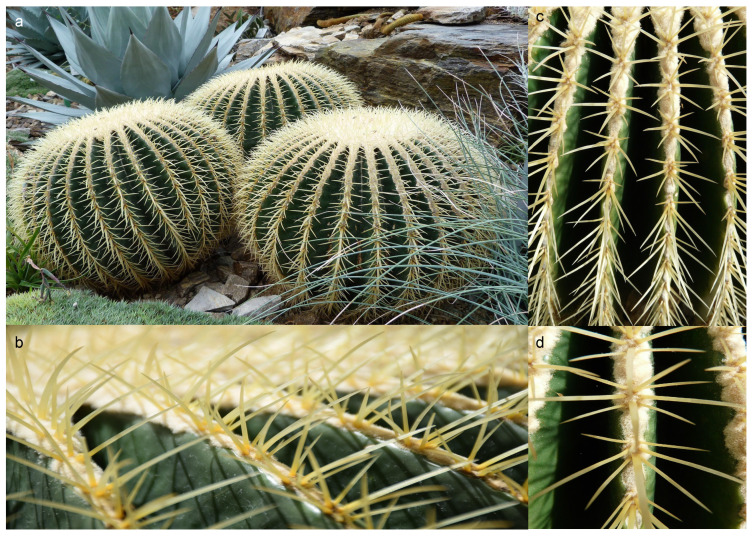
(**a**) *Echinocactus grusonii* in a wild natural setting, (**b**) arrangement of spines on the sides of cactus, (**c**) arrangement of spines on the top of cactus, and (**d**) magnification to show particulars of the spine structure and arrangement (https://pixabay.com/images/search/echinocactus%20grusonii/ (accessed on 18 February 2025)).

In contrast, cactus spines were investigated in the literature related to their ability to collect fog [86], directionally transport water [87,88], and with reference to their efficacy in the water-in-oil emulsion separation [89]. Moreover, the hierarchical structure and mechanics of plant materials were studied in [90], while climbing plants were used as models for bio-inspired growing robots able to attach to different substrates [91]. Again, the structure and morphology of cladodes and spines of Opuntia ficus-indica were investigated in [92], and structural properties of *Echinocactus grusonii* spines (see Figure 1) were explored in [93].

Nevertheless, the study of the elastic instability of *Echinocactus grusonii* spines interacting with external bodies seems to be currently lacking. Also, the biomimicry of Cactaceae has not yet been used as a source of inspiration to improve the structural design and the insertion procedure of the self-inserting intraneural interfaces.

As a consequence, in this work, the elastic stability of *Echinocactus grusonii* spines was first studied for structures unable to pierce an external body. Then, it was explored for structures which were able to pierce the external layer of a solid body. Furthermore, the effect of the insertion of a stiffer and thicker tract within the main shaft of spines was analyzed in terms of changes of their buckling threshold. Finally, biomimicry of *Echinocactus grusonii* spines was used as a novel source of inspiration for design principles and insertion procedures of self-inserting intraneural interfaces.

## 2. Methods

The structure of the cactus spine was modeled as a bi-dimensional rectilinear beam, which was loaded by an interaction force arising from the contact with an external body. The real three-dimensional shape and the elastic nature of the spine structure were accounted for through the second area moment of inertia $J_{min}$ and the longitudinal stiffness *E* (see Table 1).

### 2.1. Spine with a Single Section and Unable to Pierce the External Object

**Figure 2:**
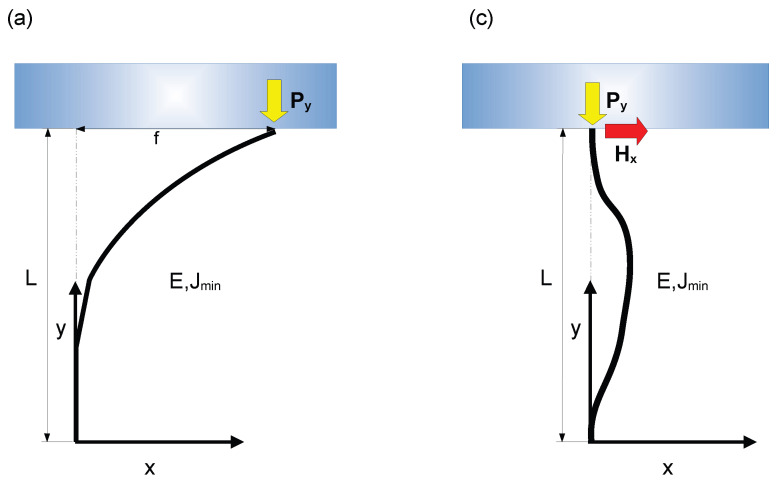

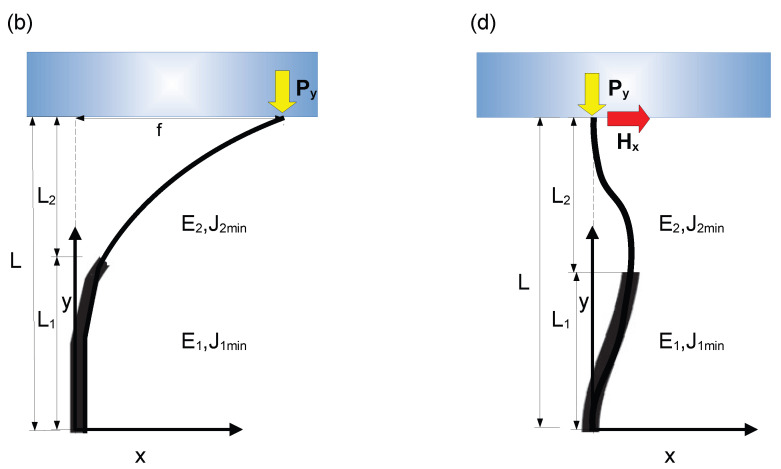
Interaction between the structure of a spine and an external solid object (in light blue). (**a**) The main shaft of the spine has a constant longitudinal stiffness *E* and a second area moment of inertia $J_{min}$(see Table 1). The tip of the spine is not able to pierce and enter the external surface of the solid object: a vertical interaction force $P_{y}$ arises, and it is represented through a vertical arrow (see Table 1). (**b**) The main shaft of the spine has two different sections with different longitudinal stiffness (i.e., $E_{1}$ and $E_{2}$), and two different second area inertial moments (i.e, $J_{1min}$ and $J_{2min}$) (see Table 1). The spine is not able to pierce the external surface of the solid object, thus a vertical interaction force $P_{y}$ (see Table 1) arises (yellow arrow). (**c**) The main shaft of the spine has a constant longitudinal stiffness *E* and a second area moment of inertia $J_{min}$ (see Table 1). The spine is able to pierce and enter the external surface of the solid object: both a vertical ($P_{y}$) and a horizontal ($H_{x}$) interaction force (see Table 1) arise (vertical yellow, and horizontal red arrows, respectively). (**d**) The main shaft of the spine has two different sections with different longitudinal stiffness $E_{1}$ and $E_{2}$, together with two different second area inertial moments $J_{1min}$ and $J_{2min}$ (see Table 1). The tip of the spine is able to pierce the external surface of the solid object, thus two interaction forces arise: a vertical one ($P_{y}$), represented with a yellow arrow, and a horizontal one ($H_{x}$), represented through a red arrow (see Table 1).

When the external object, interacting with the spine tip, was too strong to be pierced, the spine was modeled as detached from this external object (see Figure 2a). Therefore, the interaction between spine and object was modeled as a vector force parallel to the longitudinal axis of the spine $P_{y}$. To assess the amount of bending moments deforming the spine structure, the rotational equilibrium between internal and external bending moments was investigated. Thus, the vector form of this rotational equilibrium for any section of the spine was written as:

(1) $$\begin{array}{c} M_{int}+M_{ext}=0 \end{array}$$

To link this equilibrium to the physical characteristics of the spine, Equation (1) was rewritten expressing the modulus of $M_{int}$ in terms of longitudinal stiffness (*E*) and second area moment of inertia ($J_{min}$), as well as the modulus of $M_{ext}$ as a function of $P_{y}$ (see Table 1):

(2) $$\begin{array}{c} EJ_{min}x\prime \prime +P_{y}\left(x-f\right)=0 \end{array}$$

where $P_{y}$ was the modulus of the vertical interaction force vector compressing the structure, and *f* was the flectional arrow at the end of the beam (see Figure 2a and Table 1). From Equation (2), setting $\alpha^{2}=P_{y}/EJ_{min}$ (see Table 1), the standard differential equation for equilibrium of the spine was obtained as the following:

(3) $$\begin{array}{c} x\prime \prime +\alpha^{2}x=\alpha^{2}f \end{array}$$

where the symbol ″ represented the double derivative of *x* with respect to *y*. The complete solution of Equation (3) is as the following:

(4) $$\begin{array}{c} x(y)=Aexp(i\alpha y)+Bexp(-i\alpha y)+f \end{array}$$

where i∈C is the imaginary unit. However, by using the Euler rule, Equation (4) was rewritten as:

(5) $$\begin{array}{c} x(y)=(A+B)cos(\alpha y)+i(A-B)sin(\alpha y)+f. \end{array}$$

Since the value of *x*(*y*) was real, the coefficients A,B∈C were selected among complex conjugates numbers. With this restriction, the equation of the shape of the loaded spine is written as:

(6) $$\begin{array}{c} x\left(y\right)=\hat{A}cos\left(\alpha y\right)+\hat{B}sin\left(\alpha y\right)+f \end{array}$$

where $\hat{A}=A+B$ and $\hat{B}=i\left(A-B\right)$. The values of coefficients in Equation (6) were found accounting for the following boundary conditions, constraining the shape of the spine:

(7) $$\begin{array}{c} x(0)=0, \end{array}$$

(8) $$\begin{array}{c} x^{\prime }\left(0\right)=0,\; \end{array}$$

(9) $$\begin{array}{c} x(L)=f. \end{array}$$

More specifically, Equation (7) results in $\hat{A}=-f$, while Equation (8) results in $\hat{B}=0$. Therefore, Equation (6) was written as:

(10) $$\begin{array}{c} x(y)=f[1-cos(\alpha y)]. \end{array}$$

In addition, accounting for the last boundary condition in Equation (9), Equation (10) provides:

(11) $$\begin{array}{c} cos(\alpha L)=0 \end{array}$$

which, finally, results in the first buckling vertical load (see Table 1) as follows:

(12) $$\begin{array}{c} P_{y}^{*}=\frac{\pi^{2}EJ_{min}}{4L^{2}}. \end{array}$$

This buckling load was the smallest force that is able to provide an equilibrium configuration resulting in a shape different from the rectilinear one.

### 2.2. Spine with Two Different Sections with Different Stiffness and Unable to Pierce the External Object

The previous analysis modeled the cactus spine as a structure with a constant section and with a given longitudinal stiffness. However, changes in cross sectional area and stiffness could be naturally found in spines. Therefore, for sake of simplicity, two different sections were considered here (see Figure 2b). More specifically, two different tracts of the spine structure were identified as proximal (which had length $L_{1}$) and distal (which had length $L_{2}$). Thus, the total spine length was $L=L_{1}+L_{2}$. The rotational equilibrium was, then, considered, and the differential equation ruling the shape of the proximal tract of the spine (i.e, $0\leq x\leq L_{1}$) was written as the following:

(13) $$\begin{array}{c} E_{1}J_{1min}x\prime \prime +P_{y}\left(x-f\right)=0 \end{array}$$

where $E_{1}$ was the longitudinal stiffness of the proximal section and *f* was the flectional arrow at the end of the spine (i.e, for $x=L=L_{1}+L_{2}$). Similarly, the differential equation ruling the shape of the distal tract (i.e., $L_{1}\leq x\leq L_{1}+L_{2}$) was:

(14) $$\begin{array}{c} E_{2}J_{2min}x\prime \prime +P_{y}\left(x-f\right)=0 \end{array}$$

where $E_{2}$ was the longitudinal stiffness of the distal section and *f* was the flectional arrow at the end of the spine (i.e, for $x=L=L_{1}+L_{2}$). Setting $\alpha_{1}^{2}=\frac{P_{y}}{E_{1}J_{1min}}$ and $\alpha_{2}^{2}=\frac{P_{y}}{E_{2}J_{2min}}$ (see Table 1), the complete solution of Equation (13) is written as:

(15) $$\begin{array}{c} x_{1}\left(y\right)=c_{1}sin\left(\alpha_{1}y\right)+c_{2}cos\left(\alpha_{1}y\right)+f, \end{array}$$

while the complete solution of Equation (14) was:

(16) $$\begin{array}{c} x_{2}\left(y\right)=c_{3}sin\left(\alpha_{2}y\right)+c_{4}cos\left(\alpha_{2}y\right)+f. \end{array}$$

The solution should satisfy the following boundary conditions:

(17) $$\begin{array}{c} x_{1}\left(0\right)=0, \end{array}$$

(18) $$\begin{array}{c} x_{1}^{\prime }\left(0\right)=0, \end{array}$$

(19) $$\begin{array}{c} x_{1}\left(L_{1}\right)=x_{2}\left(L_{1}\right),\; \end{array}$$

(20) $$\begin{array}{c} x_{1}^{\prime }\left(L_{1}\right)=x_{2}^{\prime }\left(L_{1}\right),\; \end{array}$$

(21) $$\begin{array}{c} x_{2}\left(L\right)=f. \end{array}$$

The four constants $c_{1},c_{2},c_{3},c_{4}\in R$ are found through Equations (17)–(19), together with Equation (20), and result in the following:

(22) $$c_{1}=0,$$

(23) $$c_{2}=-f,$$

(24) $$c_{3}=-f\frac{\alpha_{2}cos\left(L_{1}\alpha_{1}\right)sin\left(L_{1}\alpha_{2}\right)-\alpha_{1}sin\left(L_{1}\alpha_{1}\right)cos\left(L_{1}\alpha_{2}\right)}{\alpha_{2}},$$

(25) $$c_{4}=-f\frac{\alpha_{1}sin\left(L_{1}\alpha_{1}\right)sin\left(L_{1}\alpha_{2}\right)+\alpha_{2}cos\left(L_{1}\alpha_{1}\right)cos\left(L_{1}\alpha_{2}\right)}{\alpha_{2}}.$$

Thus, the shape of the spine for the first tract ($0\leq x\leq L_{1}$) is:

(26) $$\begin{array}{c} x_{1}\left(y\right)=f\left[1-cos\left(\alpha_{1}y\right)\right], \end{array}$$

while for the second tract ($L_{1}\leq x\leq L_{2}+L_{1}$) it is:

(27) $$\begin{array}{cc} x_{2}\left(y\right)= & \frac{f}{\alpha_{2}}\{\left[\alpha_{1}sin\left(L_{1}\alpha_{1}\right)cos\left(L_{1}\alpha_{2}\right)-\alpha_{2}cos\left(L_{1}\alpha_{1}\right)sin\left(L_{1}\alpha_{2}\right)\right]sin\left(\alpha_{2}y\right)-\\ \; & -\left[\alpha_{1}sin\left(L_{1}\alpha_{1}\right)sin\left(L_{1}\alpha_{2}\right)+\alpha_{2}cos\left(L_{1}\alpha_{1}\right)cos\left(L_{1}\alpha_{2}\right)\right]cos\left(\alpha_{2}y\right)+\alpha_{2}\}. \end{array}$$

Moreover, the boundary condition Equation (21) is used to determine the first buckling load through:

(28) $$\begin{array}{c} tan\left(L_{1}\alpha_{1}\right)tan\left(L_{2}\alpha_{2}\right)=\frac{\alpha_{1}}{\alpha_{2}}. \end{array}$$

Equation (28) expresses the instability condition as a function of $\alpha_{1}$ and $\alpha_{2}$, and then as a function of the physical characteristics of the spine (see Table 1).

### 2.3. Spine with a Single Section Which Is Able to Pierce an External Object

The tip of the spine is often sharp enough to pierce the external layer of a solid object. In this case, the spine tip was able to perform rotations about the point of contact. As a consequence, together with the vertical force $P_{y}$ a further force $H_{x}$ arose (see Table 1), which prevented the spine tip from the horizontal motion (see Figure 2c). Then, the rotational equilibrium for any section of the spine is written as a function of both physical characteristics and external forces as the following:

(29) $$\begin{array}{c} EJ_{min}x\prime \prime +P_{y}x-H_{x}\left(L-y\right)=0. \end{array}$$

If $\alpha^{2}=\frac{P_{y}}{EJ_{min}}$ and $\omega =\frac{H_{x}}{EJ_{min}}$ (see Table 1), Equation (29) is written as:

(30) $$\begin{array}{c} x\prime \prime +\alpha^{2}x-\omega \left(L-y\right)=0. \end{array}$$

The general solution of Equation (30) is written as:

(31) $$\begin{array}{c} x\left(y\right)=Aexp\left(i\alpha y\right)+Bexp\left(-i\alpha y\right)+\frac{\omega }{\alpha^{2}}\left(L-y\right) \end{array}$$

and through the Euler rule Equation (31) is written as:

(32) $$\begin{array}{c} x\left(y\right)=\hat{A}sin\left(\alpha y\right)+\hat{B}cos\left(\alpha y\right)+\frac{\omega }{\alpha^{2}}\left(L-y\right) \end{array}$$

where $\hat{A}=A+B$ and $\hat{B}=i\left(A-B\right)$. Following Equation (32) one can get the following:

(33) $$\begin{array}{c} x^{\prime }\left(y\right)=\alpha \hat{A}cos\left(\alpha y\right)-\alpha \hat{B}sin\left(\alpha y\right)-\frac{\omega }{\alpha^{2}}. \end{array}$$

In this case, the boundary conditions constraining the spine shape are the following:

(34) $$\begin{array}{c} x(0)=0, \end{array}$$

(35) $$\begin{array}{c} x^{\prime }\left(0\right)=0,\; \end{array}$$

(36) $$\begin{array}{c} x(L)=0. \end{array}$$

Therefore, accounting for Equations (32) and (33) together with Equations (34) and (35), the value of constants are determined as $\hat{A}=\frac{\omega }{\alpha^{3}}$ and $\hat{B}=-\frac{\omega L}{\alpha^{2}}$. Thus, Equation (32) is finally rewritten as the following:

(37) $$\begin{array}{c} x\left(y\right)=\frac{\omega }{\alpha^{3}}sin\left(\alpha y\right)-\frac{\omega L}{\alpha^{2}}cos\left(\alpha y\right)+\frac{\omega }{\alpha^{2}}\left(L-y\right). \end{array}$$

Moreover, Equation (37) through Equation (36) leads to:

(38) $$\begin{array}{c} \frac{\omega }{\alpha^{3}}\left[sin\left(\alpha L\right)-\alpha Lcos\left(\alpha L\right)\right]=0. \end{array}$$

As a consequence, the instability condition is written as:

(39) $$\begin{array}{c} tan(\alpha L)=\alpha L. \end{array}$$

As suitable approximation of the first solution of Equation (39) (error $2.12\times 10^{-4}$) is

(40) $$\begin{array}{c} \alpha L=1.4303\pi \end{array}$$

which results in the first buckling load as follows:

(41) $$\begin{array}{c} P_{y}^{*}={\left(1.4303\pi \right)}^{2}\frac{EJ_{min}}{L^{2}}≃2\pi^{2}\frac{EJ_{min}}{L^{2}}. \end{array}$$

### 2.4. Spine with Two Different Sections Able to Pierce an External Object

Also in this case, the spine structure was modeled as interacting with the external object (see Figure 2d). Again, the spine tip was able to perform rotations about the point of contact, thus, together with the vertical force $P_{y}$, a horizontal force $H_{x}$ arose, which prevented the spine tip from the horizontal motion (see Table 1). In this case, within the proximal tract 0≤y≤L1, the rotational equilibrium for any section of the spine was:

(42) $$\begin{array}{c} E_{1}J_{1min}x_{1}^{\prime \prime }+P_{y}x_{1}-H_{x}\left(L-y\right)=0 \end{array}$$

where $E_{1}$ and $J_{1min}$ were the longitudinal stiffness and the second area moment of inertia for the first section. On the contrary, within the second tract $L_{1}\leq y\leq L_{2}+L_{1}$, the rotational equilibrium equation for any section of the spine was:

(43) $$\begin{array}{c} E_{2}J_{2min}x_{2}^{\prime \prime }+P_{y}x_{2}-H_{x}\left(L-y\right)=0 \end{array}$$

where $E_{2}$ and $J_{2min}$ were the longitudinal stiffness and the second area moment of inertia for the second section. Also in this case, if $\alpha_{1}^{2}=\frac{P_{y}}{E_{1}J_{1min}}$, and $\alpha_{2}^{2}=\frac{P_{y}}{E_{2}J_{2min}}$ (see Table 1), while $\omega_{1}=\frac{H_{x}}{E_{1}J_{1min}}$, and $\omega_{2}=\frac{H_{x}}{E_{2}J_{2min}}$ (see Table 1). Equations (42) and (43) are, then, rewritten as:

(44) $$\begin{array}{c} x_{1}^{\prime \prime }+\alpha_{1}x_{1}-\omega_{1}\left(L-y\right)=0 \end{array}$$

and

(45) $$\begin{array}{c} x_{2}^{\prime \prime }+\alpha_{2}x_{2}-\omega_{2}\left(L-y\right)=0. \end{array}$$

The complete solutions are:

(46) $$\begin{array}{c} x_{1}\left(y\right)=c_{1}sin\left(\alpha_{1}y\right)+c_{2}cos\left(\alpha_{1}y\right)+\frac{\omega_{1}}{\alpha_{1}^{2}}\left(L-y\right) \end{array}$$

and

(47) $$\begin{array}{c} x_{2}\left(y\right)=c_{3}sin\left(\alpha_{2}y\right)+c_{4}cos\left(\alpha_{2}y\right)+\frac{\omega_{2}}{\alpha_{2}^{2}}\left(L-y\right) \end{array}$$

for the proximal and the distal tract, respectively. In addition, the first derivatives with respect to y are:

(48) $$\begin{array}{c} x_{1}^{\prime }\left(y\right)=c_{1}\alpha_{1}cos\left(\alpha_{1}y\right)-c_{2}\alpha_{1}sin\left(\alpha_{1}y\right)-\frac{\omega_{1}}{\alpha_{1}^{2}} \end{array}$$

and

(49) $$\begin{array}{c} x_{2}^{\prime }\left(y\right)=c_{3}\alpha_{2}cos\left(\alpha_{2}y\right)-c_{4}\alpha_{2}sin\left(\alpha_{2}y\right)-\frac{\omega_{2}}{\alpha_{2}^{2}}. \end{array}$$

The boundary conditions constraining the spine shape are the following:

(50) $$\begin{array}{c} x_{1}\left(0\right)=0, \end{array}$$

(51) $$\begin{array}{c} x_{1}^{\prime }\left(0\right)=0, \end{array}$$

(52) $$\begin{array}{c} x_{1}\left(L_{1}\right)=x_{2}\left(L_{2}\right),\; \end{array}$$

(53) $$\begin{array}{c} x_{1}^{\prime }\left(L_{1}\right)=x_{2}^{\prime }\left(L_{1}\right),\; \end{array}$$

(54) $$\begin{array}{c} x_{2}\left(L\right)=0. \end{array}$$

Therefore, accounting for Equations (46) and (48) together with Equations (50) and (51), the value of constants are determined as:

(55) $$c_{1}=\frac{\omega_{1}}{\alpha^{3}}$$

(56) $$c_{2}=-\frac{\omega_{1}L}{\alpha^{2}}$$

Thus, for the first tract ($0\leq x\leq L_{1}$) the shape is:

(57) $$\begin{array}{c} x_{1}\left(y\right)=\frac{\omega_{1}}{\alpha_{1}^{3}}sin\left(\alpha_{1}y\right)-\frac{\omega_{1}L}{\alpha_{1}^{2}}cos\left(\alpha_{1}y\right)+\frac{\omega_{1}}{\alpha_{1}^{2}}\left(L-y\right). \end{array}$$

The other constants are found through the boundary conditions in Equations (52) and (53). As a consequence, the shape of the spine for the distal tract ($L_{1}\leq y\leq L_{2}+L_{1}$) is ruled through Equation (47), where the constants $c_{3}$ and $c_{4}$ result in:

(58) $$\begin{array}{cc} c_{3}= & \frac{L_{1}sin\left(L_{1}\alpha_{2}\right)\omega_{2}}{\alpha_{2}^{2}}-\frac{Lsin\left(L_{1}\alpha_{2}\right)\omega_{2}}{\alpha_{2}^{2}}+\frac{cos\left(L_{1}\alpha_{2}\right)\omega_{2}}{\alpha_{2}^{3}}+\\ \; & +\frac{Lsin\left(L_{1}\alpha_{2}+L_{1}\alpha_{1}\right)\omega_{1}}{2\alpha_{1}\alpha_{2}}-\frac{Lsin\left(L_{1}\alpha_{2}+L_{1}\alpha_{1}\right)\omega_{1}}{2\alpha_{1}^{2}}+\\ \; & +\frac{cos\left(L_{1}\alpha_{2}+L_{1}\alpha_{1}\right)\omega_{1}}{2\alpha_{1}^{2}\alpha_{2}}-\frac{cos\left(L_{1}\alpha_{2}+L_{1}\alpha_{1}\right)\omega_{1}}{2\alpha_{1}^{3}}-\\ \; & -\frac{Lsin\left(L_{1}\alpha_{2}-L_{1}\alpha_{1}\right)\omega_{1}}{2\alpha_{1}\alpha_{2}}-\frac{Lsin\left(L_{1}\alpha_{2}-L_{1}\alpha_{1}\right)\omega_{1}}{2\alpha_{1}^{2}}+\\ \; & +\frac{cos\left(L_{1}\alpha_{2}-L_{1}\alpha_{1}\right)\omega_{1}}{2\alpha_{1}^{2}\alpha_{2}}+\frac{cos\left(L_{1}\alpha_{2}-L_{1}\alpha_{1}\right)\omega_{1}}{2\alpha_{1}^{3}}-\\ \; & -\frac{L_{1}sin\left(L_{1}\alpha_{2}\right)\omega_{1}}{\alpha_{1}^{2}}+\frac{Lsin\left(L_{1}\alpha_{2}\right)\omega_{1}}{\alpha_{1}^{2}}-\frac{cos\left(L_{1}\alpha_{2}\right)\omega_{1}}{\alpha_{1}^{2}\alpha_{2}} \end{array}$$

and

(59) $$\begin{array}{cc} c_{4}= & -\frac{sin\left(L_{1}\alpha_{2}\right)\omega_{2}}{\alpha_{2}^{3}}+\frac{L_{1}cos\left(L_{1}\alpha_{2}\right)\omega_{2}}{\alpha_{2}^{2}}-\frac{Lcos\left(L_{1}\alpha_{2}\right)\omega_{2}}{\alpha_{2}^{2}}-\\ \; & -\frac{Lsin\left(L_{1}\alpha_{1}\right)sin\left(L_{1}\alpha_{2}\right)\omega_{1}}{\alpha_{1}\alpha_{2}}-\frac{cos\left(L_{1}\alpha_{1}\right)sin\left(L_{1}\alpha_{2}\right)\omega_{1}}{\alpha_{1}^{2}\alpha_{2}}+\\ \; & +\frac{sin\left(L_{1}\alpha_{2}\right)\omega_{1}}{\alpha_{1}^{2}\alpha_{2}}+\frac{sin\left(L_{1}\alpha_{1}\right)cos\left(L_{1}\alpha_{2}\right)\omega_{1}}{\alpha_{1}^{3}}+\frac{Lcos\left(L_{1}\alpha_{2}\right)\omega_{1}}{\alpha_{1}^{2}}-\\ \; & -\frac{Lcos\left(L_{1}\alpha_{1}\right)cos\left(L_{1}\alpha_{2}\right)\omega_{1}}{\alpha_{1}^{2}}-\frac{L_{1}cos\left(L_{1}\alpha_{2}\right)\omega_{1}}{\alpha_{1}^{2}}. \end{array}$$

The instability condition is found through Equation (54) and results in:

(60) $$\begin{array}{cc} \; & -\{\{\{\left[L_{2}\alpha_{1}^{3}\alpha_{1}2sin\left(L_{1}\alpha_{2}\right)-\alpha_{1}^{3}cos\left(L_{1}\alpha_{2}\right)\right]sin\left[\left(L_{2}+L_{1}\right)\alpha_{2}\right]+\\ \; & +\left[\alpha_{1}^{3}sin\left(L_{1}\alpha_{2}\right)+L_{2}\alpha_{1}^{3}\alpha_{2}cos\left(L_{1}\alpha_{2}\right)\right]cos\left[\left(L_{2}+L_{1}\right)\alpha_{2}\right)\}\omega_{2}+\\ \; & +\{\{\left[-sin\left(L_{1}\alpha_{1}\right)+\left(L_{2}+L_{1}\right)\alpha_{1}cos\left(L_{1}\alpha_{1}\right)-L_{2}\alpha_{1}\right]\alpha_{2}^{3}sin\left(L_{1}\alpha_{2}\right)+\\ \; & +\left[\left(-L_{2}-L_{1}\right)\alpha_{1}^{2}sin\left(L_{1}\alpha_{1}\right)-\alpha_{1}cos\left(L_{1}\alpha_{1}\right)+\alpha_{1}\right]\alpha_{2}^{2}cos\left(L_{1}\alpha_{2}\right)\}sin\left[\left(L_{2}+L_{1}\right)\alpha_{2}\right]+\\ \; & +\{\left[\left(L_{2}+L_{1}\right)\alpha_{1}^{2}sin\left(L_{1}\alpha_{1}\right)+\alpha_{1}cos\left(L_{1}\alpha_{1}\right)-\alpha_{1}\right]\alpha_{2}^{2}sin\left(L_{1}\alpha_{2}\right)+\\ \; & +\left[-sin\left(L_{1}\alpha_{1}\right)+\left(L_{2}+L_{1}\right)\alpha_{1}cos\left(L_{1}\alpha_{1}\right)-L_{2}\alpha_{1}\right]\alpha_{2}^{3}cos\left(L_{1}\alpha_{2}\right)\}cos\left[\left(L_{2}+L_{1}\right)\alpha_{2}\right]\}\omega_{1}\}\\ \; & /(\alpha_{1}^{3}\alpha_{2}^{3})\}=0. \end{array}$$

For sake of simplicity, the cactus spine is modeled as beam-like structures with a circular cross sectional area with radius *R*. As a consequence, the explicit form of the minimum second area moment of inertia is as follows:

(61) $$J_{min}=\frac{\pi }{4}R^{4}.$$

To easy the comparison between results of Equations (12), (28), (41) and (60) the first buckling load is expressed as:

(62) $$P_{y}^{*}=N^{2}\frac{E_{i}R_{i}^{4}}{L^{2}}$$

where *N* is derived from the instability conditions related to the geometry of the beam-like structure. Moreover, for a spine with a single section, $E_{i}$ is the longitudinal stiffness of the whole structure (i.e., $E_{i}=E$). On the contrary, $E_{i}$ is the longitudinal stiffness of the distal section (i.e., $E_{i}=E_{2}$), for a spine with two different sections. Similarly, $R_{i}$ is the radius of the whole structure (i.e., $R_{i}=R$) for a spine with a single section, while it is the radius of the distal tract, for a spine with two sections (i.e., $R_{i}=R_{2}$). In any case, *L* is the total length of the structure. However, Equation (62) is theoretical, then all quantities are assumed to be exactly known. Nevertheless, experimental quantities are known only with errors. Therefore, the error propagation in Equation (62) is calculated in Appendix A to quantify the influence of experimental errors about $E_{i}$, $R_{i}$ and $L_{i}$ on the first buckling load determination.

## 3. Results

### 3.1. Buckling of a Spine with a Single Section Unable to Pierce the Surface of the External Object

**Figure 3:**
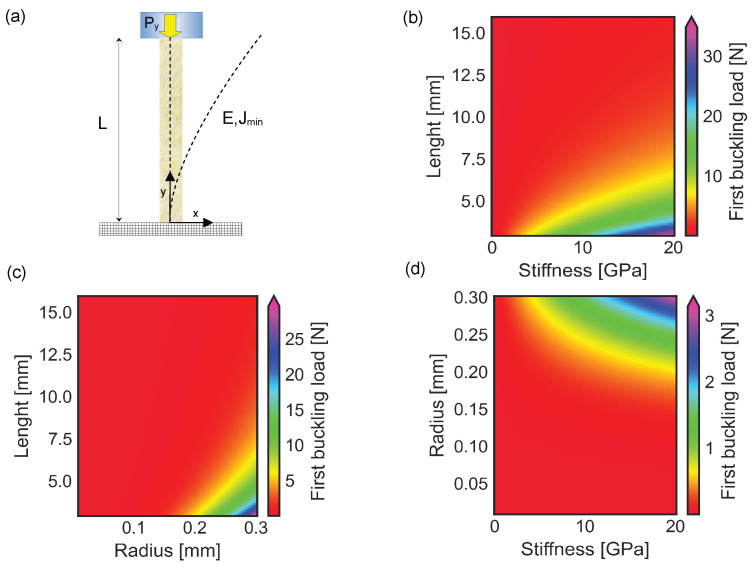
Buckling of a spine with constant circular cross sectional area: (**a**) Frontal view with dashed lines representing the barycentric line of the undeformed structure (straight line) and the barycentric line of the structure deformed under the action of the critical buckling load $P_{y}$ (curved dashed line), respectively. (**b**) Plot of changes in first buckling load for spines with a length ranging between 3 mm and 15 mm, stiffness up to 20 GPa, and radius R=0.3 mm. (**c**) Plot of changes in first buckling load for spines with E=17 GPa, length between 3 mm and 15 mm and radius up to 0.3 mm. (**d**) Plot of changes in first buckling load for spines with L=10 mm, radius up to 0.3 mm and stiffness up to 20 GPa.

The interaction between the spine structure and the external object was modeled through a contact force compressing the spine along its barycentric line. In addition, the force was applied at the center of the cross sectional area, which was assumed to be circular. The compression force increased up to the first buckling load, for which the structure suddenly deformed (see Figure 3a, dashed line). The changes in stiffness and length of the spine influenced the value of the first buckling load, which was ruled by Equation (12). In particular, the elastic instability was reached for forces lower than 10 N in spines with a stiffness ranging between 5 and 20 GPa, a length between 7.5 mm and 15 mm, and a constant radius R = 0.3 mm (see Figure 3b). In contrast, the first buckling load rapidly increased up to 20 N, when the structure length decreased below 7 mm. Similarly, the first buckling load was lower than 5 N for thin structures with a radius smaller than R=0.2 mm, a length larger than 5 mm, and a constant stiffness E=17 GPa (see Figure 3c). The increase of the radius up to R=0.3 mm was able to raise the first buckling load up to 20–25 N. Finally, for structures with a total length L=10 mm, the instability was reached for forces lower than 1 N, a cross sectional radius smaller than R=0.2 mm, and a stiffness up to 20 GPa (see Figure 3d).

### 3.2. Buckling of a Spine Unable to Pierce the Surface of an External Object and Having a Double Section

**Figure 4:**
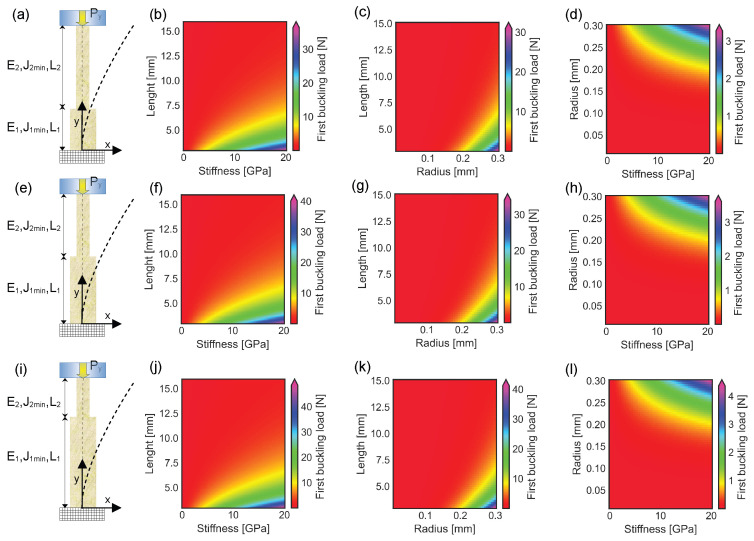
Plots of first buckling load modification for different spines unable to pierce the external surface of an object. For sake of simplicity, all the cross sectional areas are supposed to be circular: (**a**) Frontal view of a spine with two different sections. The radius of the proximal section is $R_{1}=4/3R_{2}$, while its stiffness is $E_{1}=4/3E_{2}$, where $R_{2}$ and $E_{2}$ are the radius and the stiffness of the distal section. In this case, the length of the proximal section is half of the distal one ($L_{1}=0.5L_{2}$). The dashed lines represent the barycentric line of the undeformed structure (straight line) and the barycentric line of the structure deformed under the action of the critical buckling load $P_{y}^{*}$ (curved dashed line). (**b**) First buckling load modification for spines with $R_{2}=0.3$ mm, a stiffness up to 20 GPa, and a total length ($L=L_{1}+L_{2}$) varying between 3 mm and 15 mm. (**c**) Change in first buckling load for spines with a distal radius $R_{2}$ up to 0.3 mm, a total length between 3 mm and 15 mm, and a constant distal stiffness of $E_{2}=17$ GPa. (**d**) First buckling load modification for spines with a distal stiffness $E_{2}$ up to 20 GPa, a radius $R_{2}$ up to 0.3 mm, and a constant total length L=10 mm. (**e**) Frontal view of spine structures with two different sections: the length of both proximal and distal sections of the structure is the same ($L_{2}$ = $L_{1}$), while $R_{1}=4/3R_{2}$ and $E_{1}=4/3E_{2}$. The dashed lines represent the barycentric line of the undeformed structure (straight line) and the barycentral line of the structure deformed under the action of the critical buckling load $P_{y}^{*}$ (curved dashed line). (**f**) Change in first buckling load for spines with $R_{2}=0.3$ mm, a stiffness up to 20 GPa, and a total length varying between 3 mm and 15 mm. (**g**) First buckling load modification for a distal radius $R_{2}$ up to 0.3 mm, a total length *L* varying between 3 mm and 15 mm, and a constant distal stiffness of $E_{2}=17$ GPa. (**h**) First buckling load variation for a distal stiffness $E_{2}$ up to 20 GPa, a radius $R_{2}$ up to 0.3 mm, and a constant length L=10 mm. (**i**) Frontal view of a spine structure with two different sections: the length of the proximal section is two times the length of the distal one ($L_{1}=2L_{2}$), while $R_{1}=4/3R_{2}$ and $E_{1}=4/3E_{2}$. The dashed lines represent the undeformed barycentric line (straight line) and the barycentric line of the structure deformed under the action of the critical buckling load $P_{y}^{*}$ (curved dashed line). (**j**) Change in first buckling load for structures with $R_{2}=0.3$ mm, a distal stiffness up to 20 GPa, and a total length varying between 3 mm and 15 mm. (**k**) First buckling load modification for spines with a distal radius $R_{2}$ up to 0.3 mm, a length *L* varying between 3 mm and 15 mm, and a constant distal stiffness of $E_{2}=17$ GPa. (**l**) First buckling load variation for a distal stiffness $E_{2}$ up to 20 GPa, a distal radius $R_{2}$ up to 0.3 mm, and a constant total length L=10 mm.

The main shaft of the spine was deformed under the action of a compressive force even for a variable cross sectional area. An increase in both cross sectional radius and stiffness was investigated in Figure 4a, where the proximal reinforced section was 1/3 of the total length. In this case, the first buckling load was lower than 10 N, for spines with a length between 7.5 mm and 15 mm, a stiffness up to 20 GPa, and a radius of the distal section $R_{2}=0.3$ mm (Figure 4b). The first buckling load was lower than 10 N, for spines longer than 5 mm, with a distal section stiffness $E_{2}=17$ GPa, and a radius up to 0.2 mm (Figure 4c). Furthermore, the buckling threshold was lower 1 N, for spines L=10 mm long, with a stiffness up to 20 GPa, and a distal radius up to 0.2 mm (Figure 4d). When the length of the proximal reinforced section was half of the whole structure length, the first buckling load was below 15 N, if the structure stiffness was up to 20 GPa, its length above 7.5 mm, and the distal radius $R_{2}=0.3$ mm (Figure 4f). Again, the first buckling load was lower than 10 N, for a cross sectional radius up to 0.3 mm, a length above 7.5 mm, and a distal stiffness of $E_{2}$ = 17 GPa (Figure 4g). If the spine stiffness was up to 20 GPa, the first buckling threshold was lower than 1 N, for a distal cross sectional radius up to 0.15 mm, and a whole length L=10 mm (Figure 4h). Analogously, when the length of the proximal reinforced section was 2/3 of the total spine length, the first buckling load was below 10 N, for a stiffness up to 20 GPa, a length greater than 7.5 mm, and a constant distal radius $R_{2}=0.3$ mm (Figure 4j). Similarly, for spines with a distal stiffness $E_{2}=17$ GPa, the first buckling load was lower than 10 N, if the distal cross sectional radius was up to 0.3 mm, and the length larger than 7.5 mm (Figure 4k). Finally, for structures L=10 mm long, the first buckling load was lower than 1 N, for a cross sectional radius up to 0.15 mm, and a stiffness up to 20 GPa (Figure 4l).

### 3.3. Buckling of a Spine Able to Pierce the External Object and Having a Constant Section

**Figure 5:**
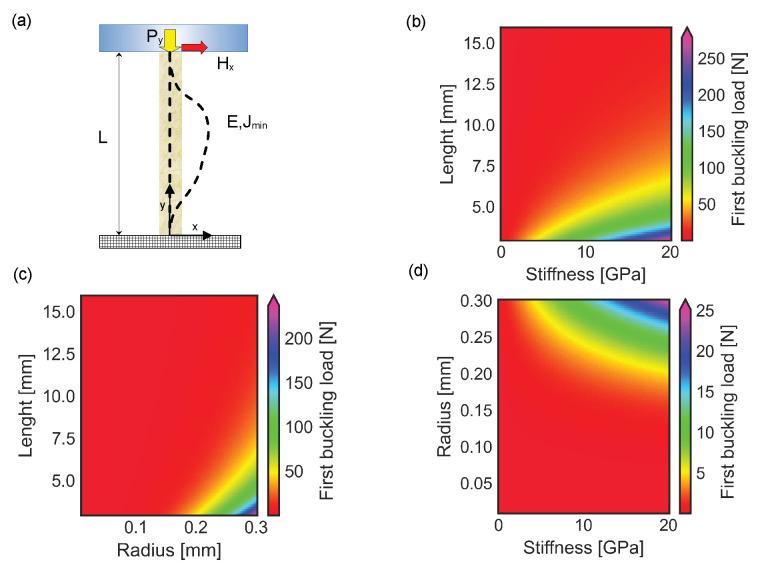
First buckling load modification for spines with constant circular cross sectional area: (**a**) Frontal view with the dashed lines representing the barycentric line of the undeformed structure (straight line) and the barycentric line of the structure deformed under the action of the critical buckling load $P_{y}^{*}$ (curved dashed line), respectively. (**b**) Change in first buckling load for spines with a length ranging between 3 mm and 15 mm, a stiffness up to 20 GPa, and a radius of 0.3 mm. (**c**) First buckling load modification for spines with constant stiffness E = 17 GPa, a length between 3 mm and 15 mm, and radius up to 0.3 mm. (**d**) Change in first buckling load for spines with constant length L = 10 mm, a radius up to 0.3 mm, and a stiffness ranging up to 20 GPa.

When the tip of the spine was able to pierce the external surface of a solid object, the interaction was modeled through two forces: a vertical compression force $P_{y}$ and a horizontal reaction force $H_{x}$ (see Figure 5a). Also in this case, the compression force increased up to the first buckling load, when the structure suddenly deformed (see Figure 5a dashed line). The value of the first buckling load, which in this case was ruled by Equation (41), varied with changes in both length and stiffness of the structure. More specifically, for a constant cross sectional radius R=0.3 mm, the first buckling load was smaller than 50 N for structures with a length ranging between 7.5 mm and 15 mm, and a stiffness up to 20 GPa (Figure 5b). Similarly, for a constant stiffness E=17 GPa, the first buckling load was lower than 10 N for structures with a length between 7.5 mm and 15 mm and cross sectional radius up to R=0.3 mm (Figure 5c). Finally, the value of the first buckling load was lower than 5 N for spines with a length of L=10 mm, a cross sectional radius up to 0.2 mm, and a stiffness up to 20 GPa (see Figure 5d).

### 3.4. Buckling of Spines Having with Two Different Sections and Able to Pierce the External Surface of an Object

**Figure 6:**
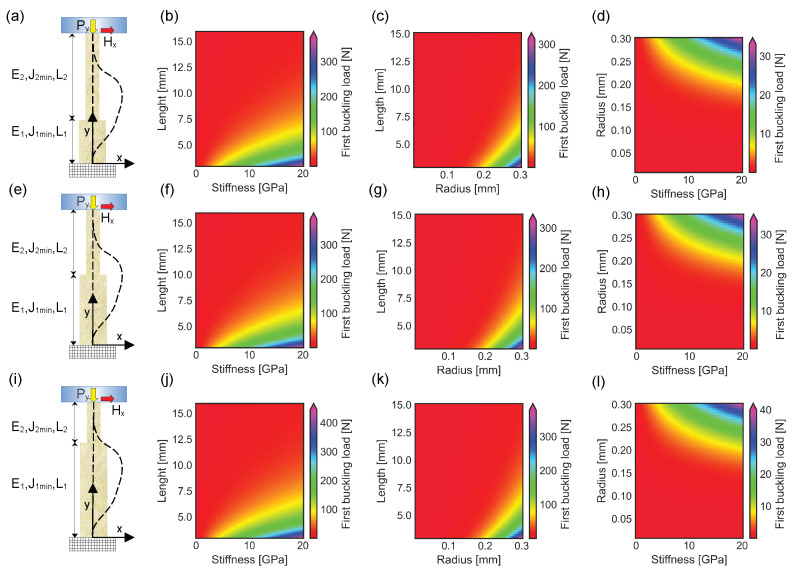
Plots of first buckling load modification for different spines able to pierce the external surface of an object. For sake of simplicity, all the cross sectional areas are supposed to be circular: (**a**) Frontal view of spine structures with two different sections. The radius of the proximal section is $R_{1}=4/3R_{2}$, while the stiffness is $E_{1}=4/3E_{2}$, where $R_{2}$ and $E_{2}$ are the radius and the stiffness of the distal part. In this case, the length of the proximal section is the half of the distal one ($L_{1}=0.5L_{2}$). Dashed lines represent the barycentric line of the undeformed structure (straight line) and the barycentric line of the structure deformed under the action of the critical buckling load $P_{y}^{*}$ (curved dashed line) together with the horizontal reaction force. (**b**) Change in first buckling load for spines with $R_{2}=0.3$ mm, a stiffness up to 20 GPa, and a total length ($L=L_{1}+L_{2}$) varying between 3 mm and 15 mm. (**c**) First buckling load modification for spines with a distal radius $R_{2}$ up to 0.3 mm, a total length varying between 3 mm and 15 mm, and a constant distal stiffness of $E_{2}=17$ GPa. (**d**) First buckling load variation for structures with a distal stiffness $E_{2}$ up to 20 GPa, a distal radius $R_{2}$ up to 0.3 mm, and a constant total length L=10 mm. (**e**) Frontal view of spines with two different sections: the length of both proximal and distal sections is the same ($L_{2}$=$L_{1}$), while $R_{1}=4/3R_{2}$ and $E_{1}=4/3E_{2}$. The dashed lines represent the barycentric line of the undeformed structure (straight line) and the barycentric line of the structure deformed under the action of the critical buckling load $P_{y}^{*}$ (curved dashed line) together with the horizontal reaction force. (**f**) Change in first buckling load for spines with $R_{2}=0.3$ mm, a distal stiffness up to 20 GPa, and a total length varying between about 3 mm and 15 mm. (**g**) First buckling load modification for spines with a distal radius $R_{2}$ up to 0.3 mm, a total length *L* varying between 3 mm and 15 mm, and a constant distal stiffness of $E_{2}=17$ GPa. (**h**) First buckling load variation for a distal stiffness $E_{2}$ up to 20 GPa, a distal radius $R_{2}$ up to 0.3 mm, and a constant total length L=10 mm. (**i**) Frontal view of spines with two different sections: the length of the proximal section is two times the length of the distal one ($L_{1}=2L_{2}$), while $R_{1}=4/3R_{2}$ and $E_{1}=4/3E_{2}$. The dashed lines represent the barycentric line of the undeformed structure (straight line) and the barycentric line of the structure deformed under the critical buckling load $P_{y}^{*}$ (curved dashed line) in presence of the resulting horizontal reaction force. (**j**) Change in first buckling load for spines with $R_{2}=0.3$ mm, a distal stiffness up to 20 GPa, and a total length varying between 3 mm and 15 mm. (**k**) First buckling load modification for a distal radius $R_{2}$ up to 0.3 mm, a total length *L* varying between 3 mm and 15 mm, and a constant distal stiffness $E_{2}=17$ GPa. (**l**) First buckling load variation for a distal stiffness $E_{2}$ up to 20 GPa, a distal radius $R_{2}$ up to 0.3 mm, and a constant total length L=10 mm.

Even in presence of a double section, when the spine structure was able to pierce the external surface of an object, the interaction was modeled through two different reactions: a vertical force $P_{y}$ and a horizontal one $H_{x}$. More specifically, when the length of the proximal section was half of the distal one (see Figure 6a), $R_{1}=4/3R_{2}$, $E_{1}=4/3E_{2}$ and $R_{2}=0.3$ mm, the value of the first buckling load $P_{y}^{*}$ was lower than 100 N, for a length varying between 7.5 mm and 15 mm, and a stiffness up to 20 GPa (see Figure 6b). The value of the first buckling load was lower than 100 N also for spines with the same length range, a distal radius $R_{2}$ up to 0.3 mm, and a distal stiffness $E_{2}=17$ GPa (see Figure 6c). On the contrary, the first buckling load was lower than 10 N when $R_{2}$ was smaller than 0.2 mm, the total length was L=10 mm, and the distal stiffness was up to 20 GPa (see Figure 6d). When the length of both the distal and the proximal sections was the same (Figure 6e), $R_{1}=4/3R_{2}$, $E_{1}=4/3E_{2}$, the value of the first buckling load was lower than 100 N for $R_{2}=0.3$ mm, for a length varying between 7.5 mm and 15 mm, and a distal stiffness up to 20 GPa (Figure 6f). Analogously, the first buckling load was lower than 100 N for the same length range, a distal radius $R_{2}$ up to 0.3 mm, and $E_{2}=17$ GPa (see Figure 6g). Moreover, the value of $P_{y}^{*}$ was lower than 10 N for a distal radius $R_{2}$ less than 0.2 mm, a distal stiffness up to 20 GPa and a total length L=10 mm (see Figure 6h). Furthermore, when the length of the distal section was half of the length of the proximal one, $R_{1}=4/3R_{2}$, $E_{1}=4/3E_{2}$ (Figure 6i), the value of the first buckling load was lower than 100 N for a length ranging between 7.5 mm and 15 mm, a stiffness $E_{2}$ up to 20 GPa and $R_{2}=0.3$ mm (Figure 6j). Similarly, the value of $P_{y}^{*}$ was lower then 100 N for the same length range, a distal radius $R_{2}$ up to 0.3 mm, and a stiffness $E_{2}=17$ GPa (see Figure 6k). Finally, the value of the first buckling load was lower than 10 N for spines with total length L=10, a distal radius $R_{2}$ smaller than 0.2 mm, and a stiffness $E_{2}$ up to 20 GPa (Figure 6l).

### 3.5. Effects of Reinforcements in Spines with a Double Section

**Figure 7:**
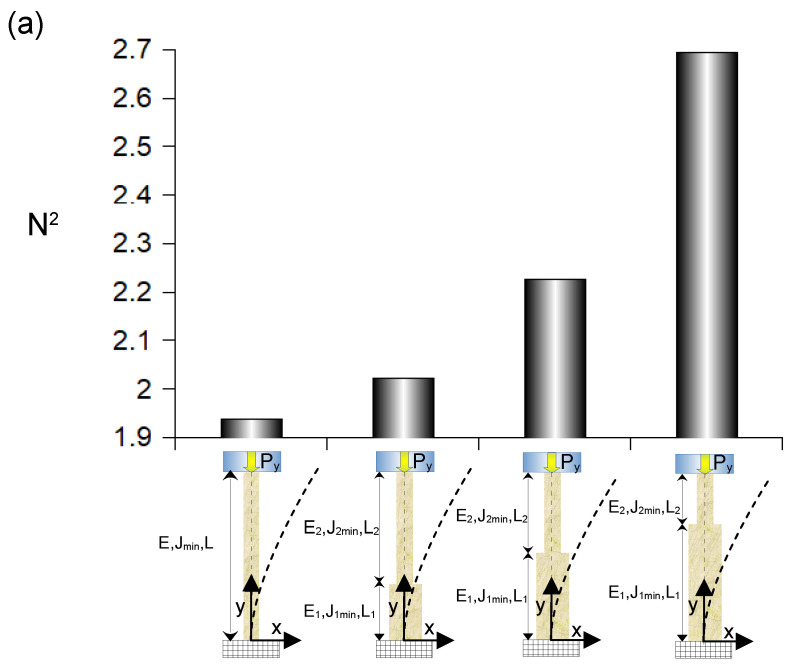

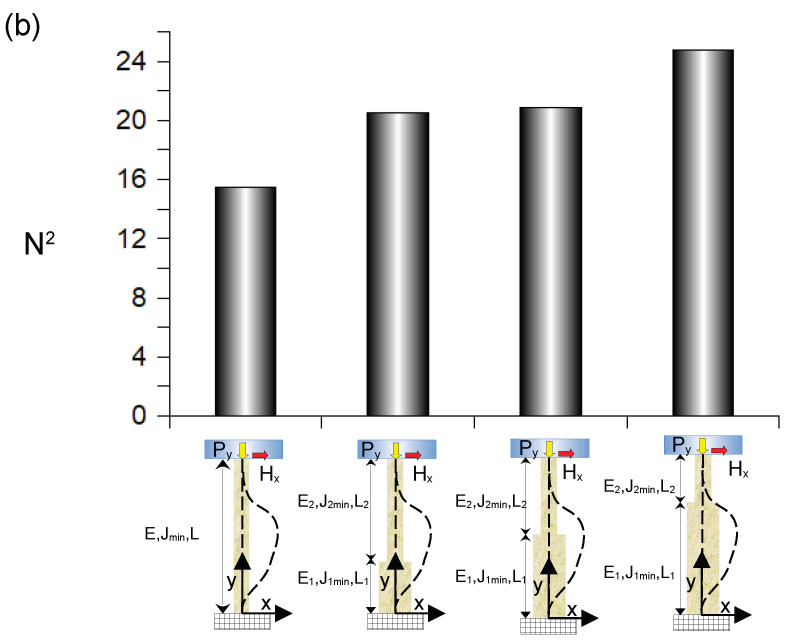
Variation of $N^{2}$ in Equation (62) for structures with an increasing length of the reinforced tract of their main shaft. (**a**) Spines unable to pierce the external surface of a solid object. For a structure with a single not reinforced section $N^{2}=1.938$, while for a structure with a reinforced tract long $L_{1}=1/3L$, $N^{2}=2.022$ (*L* = total length). Similarly, for a structure with a reinforced tract long $L_{1}=1/2L$, $N^{2}=2.227$. Analogously, for a structure with a reinforced tract long $L_{1}=2/3L$, $N^{2}=2.694$. (**b**) Spines able to pierce the external surface of a solid object and with an increasing length of the reinforced tract: for a structure with a single not reinforced section $N^{2}=15.503$, while for a structure with a reinforced tract long $L_{1}=1/3L$$N^{2}=20.472$. Moreover, for a structure with a reinforced tract long $L_{1}=1/2L$$N^{2}=20.826$. Finally, for a structure with a reinforced tract long $L_{1}=2/3L$, $N^{2}=24.730$.

The insertion of a stiffer and thicker tract in the main shaft of spines unable to pierce the external surface of a solid object resulted in the increase of their resistance to external loads. Indeed, accounting for both Equations (12) and (62), the first buckling threshold was assessed through the value of $N^{2}$. This pure number quantified the first buckling load with respect to the product between the stiffness and the fourth power of the radius divided from the square of the whole structure length (i.e., with respect to $E_{i}R_{i}^{4}/L^{2}$). For a spine with a single section, the longitudinal stiffness was $E_{i}=E$ and the radius was $R_{i}=R$, thus $N^{2}=1.938$. When the main shaft of a spine was reinforced through the insertion of a proximal tract, which was long a third of the whole length, as well as stiffer and thicker than the distal one (i.e., $E_{1}=4/3,E_{2}$, $R_{1}=4/3R_{2}$), its resistance was assessed through the first solution of Equations (28) and (62). As a consequence, the resistance to the first buckling load, for $E_{i}=E_{2}$ and $R_{i}=R_{2}$, and with reference to $E_{i}R_{i}^{4}/L^{2}$, was quantified through $N^{2}=2.022$. Similarly, when the reinforcing tract was half of the total length of the structure, Equation (62) resulted in $N^{2}=2.227$. Finally, for a reinforcing tract long two-thirds of the spine length, Equation (62) resulted in $N^{2}=2.694$ (see Figure 7a). For spines having a single section $R_{i}=R$ with stiffness $E_{i}=E$, and which were able to pierce the external surface of a solid object, Equations (41) and (62) resulted in $N^{2}=15.503$. Again, the first solution of Equation (60) together with Equation (62) resulted in $N^{2}=20.472$, when the main shaft of the spine was reinforced ($E_{i}=E_{2}$, $R_{i}=R_{2}$) with a stiffer and thicker tract long one-third of the total length (i.e., $E_{1}=4/3,E_{2}$, $R_{1}=4/3R_{2}$). Similarly, when the reinforced tract was as long as half of the total length, $N^{2}=20.876$, while, for a stiffer and thicker tract long two-thirds of the total length of the spine, $N^{2}=24.730$ (see Figure 7b).

## 4. Discussion

### 4.1. Echinocactus grusonii Strategies to Reinforce Spines

Spines are fascinating structures allowing *Echinocactus grusonii* plants to protect themselves by puncturing both animal and human tissues. In this work, the interaction between spines and an external body in contact with them was modeled through forces. More specifically, when a spine was unable to pierce the external body, the tip, under the action of the first buckling load, was able to move horizontally on the object surface to reach an equilibrium configuration different from the rectilinear one. Then, the main shaft of the spine was compressed only through a vertical force, which resulted in the formation of a flectional arrow. On the other hand, when a spine was able to pierce the external body, its tip was under the action of two different force components. Indeed, both a vertical and a horizontal component of the reaction force arose. The tip of the spine was not able to move horizontally on the external surface of the solid object, because of the presence of the horizontal component of the reaction. As a consequence, the main structure of the spine was able to equilibrate the first buckling load, but without the formation of a flectional arrow. In both cases, the main structure of the spine suddenly deformed under the action of the first buckling load to reach a novel equilibrium configuration. Equation (62) was used to compare the behaviour of spines with different geometries. This expression involved a simplified calculation of the second area moment of inertia in Equation (61), which, in its turn, implied a circular cross sectional area of the spine shafts. Although real spines could have an irregular cross sectional area, this hypothesis was acceptable since real sections were nearly elliptical and sometimes with small eccentricity [93]. In addition, *Echinocactus grusonii* spines were modeled as stiff beam-like structures characterized by their Young modulus (or by two moduli in case of two sections with different stiffness). However, experiments revealed high anisotropy in spines together with a difference between lateral and longitudinal stiffness. Nevertheless, since the elastic instability occurred longitudinally, the mean experimental value of the longitudinal Young modulus [93] was chosen as a reference in this work. Spines of *Echinocactus grusonii* could naturally present a variable cross sectional area, resulting in a proximal tract stiffer and thicker than the distal one. To model the structural effects of this reinforcement, a simplified case was studied, where only two tracts with different stiffness and thickness were present. For sake of simplicity, the stiffness of the proximal section was assumed to be the 4/3 of the distal one, while the radius of the proximal cross sectional area was assumed to be the 4/3 of the radius of the distal one. Even if real cases may be more complex, just the presence of two different sections was able to model the increase of structural performances due to a stiffer and thicker tract. The previously presented framework was able to contribute in clarifying some defense strategies of Cactaceae. Indeed, young spines are smaller and thinner, while spines in adult plants are longer and thicker. Nevertheless, the effectiveness of spines is similar in both cases, since the buckling threshold is directly proportional to the fourth power of the radius and inversely proportional to the square of the spine length. Therefore, spines are able to protect both young and adult plants, because of their almost stable resistance to compression forces. Moreover, further experimental verifications of these theoretical predictions could better assess the influence of the anisotropy of the spine material. Similarly, experiments could clarify the contribution of the geometrical variability of the spine main shaft on the quantification of the first buckling threshold.

### 4.2. Biomimicry of Echinocactus grusonii Spines Could Inspire Design Principles and Implantation Strategies of Intraneural Interfaces

Self-inserting intraneural interfaces and *Echinocactus grusonii* spines share common physical features. Indeed, self-inserting intraneural interfaces suddenly deform producing a flectional arrow under the action of a vertical buckling load, when they cannot pierce the external surface of peripheral nerves. They abruptly deform, instead, under the action of the first buckling load without the formation of any flectional arrow, when they can pierce the external surface of peripheral nerves. As a consequence, the same strategies, able to increase the first buckling threshold in spines, are effective to increase the resistance of neural interfaces to axial loads.

This analysis showed how to improve the structural resistance of self-inserting intraneural interfaces, or how to increase the first buckling threshold of structures (e.g., micro-needles) needed to insert intraneural interfaces. In particular, the previous framework was able to explicitly quantify the relationship between the cross sectional radius of self-inserting intraneural interfaces and the maximum length of their shaft. Indeed, in general, for thin self-inserting intraneural interfaces the shaft length should be minimized to maximize their buckling threshold. On the contrary, thicker structures could have longer shafts. This information may be crucial to design structures able to contact internal nervous bundles, reaching a suitable depth of insertion, which may be not negligible with respect to the dimensions of the targeted nerve.

Furthermore, the biomimicry of *Echinocactus grusonii* spines revealed that a progressive reinforcement of the main shaft through a tract with a stiffer and thicker section (i.e., 1/3, 1/2, and 2/3 of the total length of the main shaft, respectively), could increase the amount of the first buckling threshold of 4.3%, 14.9% and 39.0%, in self-inserting intraneural interface unable to pierce the external surface of nerves. Similarly, the same strategy was able to increase of 32.1%, 34.3%, and 59.5% the amount of the first buckling threshold for self-inserting intraneural interfaces having an increasing length of the reinforced tract (i.e., 1/3, 1/2, and 2/3 of the total shaft length, respectively), which were able to pierce the external surface of nerves (see Figure 4a,e,i, Figure 6a,e,i and Figure 7a,b). In addition, a further strategy was suggested by the previous analysis of the elastic instability of the *Echinocactus grusonii* spines. Indeed, comparing Equations (12) and (41) and accounting for Equation (62), a variation of the $N^{2}$ coefficient from $N^{2}=\pi^{2}/4$ to $N^{2}=2\pi^{2}$ is expected in neural interfaces with a single cross sectional area. In other words, the only presence of the horizontal force $H_{x}$ was able to increase of 8 times the amount of the first buckling threshold. As a consequence, under the same structural conditions, the ability to penetrate (and enter) the outermost layer of the nerve has a stabilizing effect on the insertion procedure. The ability to pierce the nerve can be directly achieved through an effective design of the self-inserting intraneural interface (e.g., through a quite sharp design of the tip) or through the use of a sharp external device (e.g., inserting needle) supporting the insertion within the neural tissue. Finally, it could worthy to notice that this last strategy is able to keep constant the volume of material inserted within the nerve, while the structural reinforcement of the main shaft through the increase of the proximal section increases the volume of material inserted into the nerve.

## 5. Conclusions

In this work, the piercing ability of *Echinocactus grusonii* spines was explored through the study of the interaction between their main shaft and an external object. The physics of this interaction was explored and the elastic stability of spines was considered. Two strategies to increase their structural stability were identified: the reinforcement of the main shaft through a stiffer and thicker proximal tract, and the stabilization of the global shaft deformation through the piercing of the outermost layer of the external object. Since self-inserting intraneural interfaces share structural physical features with *Echinocactus grusonii* spines, the same strategies, able to increase the first buckling threshold of spines, were proposed to increase the elastic stability of self-inserting intraneural interfaces. In particular, this study underlined that, for self-inserting intraneural interfaces with a single and constant cross section, the first buckling threshold can be increased up to 8 times only through the stabilization effect resulting from the piercing of the tissue. In addition, it was shown that a stiffer proximal tract long 2/3 of the total length was able to increase the first buckling load up to 39%, for structures unable to pierce the nerve, and up to 59% for self-inserting intraneural interfaces, which are able to pierce the external layer of nerves. Nevertheless, this last strategy may led to issues (e.g., exacerbation of the foreign body response) due to the increase of the volume of synthetic material inserted into the nerves.

## Figures and Tables

**Table 1:** Columns 1–4 show the physical nature, the symbol used in formulas, the unit of measure, and the physical description of each physical variable used to model the buckling of spines.

Physical Nature	Physical Variable	Unit of Measure	Physical Description
Stiffness (Young Modulus)	*E*	[GPa]	Extent to which a spine resists longitudinal deformations
Stiffness (Young Modulus)	$E_{1}$	[GPa]	Longitudinal stiffness proximal section
Stiffness (Young Modulus)	$E_{2}$	[GPa]	Longitudinal stiffness distal section
Second area moment	$J_{k}$	[mm]^4^	Bending stiffness along the k axis
Minimum second area moment	$J_{min}$	[mm]^4^	Minimum bending stiffness between $J_{x}$ and $J_{z}$
Minimum second area moment	$J_{1min}$	[mm]^4^	Minimum bending stiffness proximal section
Minimum second area moment	$J_{2min}$	[mm]^4^	Minimum bending stiffness distal section
Length	*L*	[mm]	Total spine length
Length	$L_{1}$	[mm]	Proximal section length
Length	$L_{2}$	[mm]	Distal section length
Length	*R*	[mm]	Cross sectional radius of spine
Length	$R_{1}$	[mm]	Proximal section radius
Length	$R_{2}$	[mm]	Distal section radius
Length	*f*	[mm]	Flectional arrow at the end of the spine
Force	$P_{y}$	[N]	Vertical interaction force
Force	$H_{x}$	[N]	Horizontal interaction force
Moment	$M_{\mathrm{int}}$	[N m]	Internal bending moment
Moment	$M_{\mathrm{ext}}$	[N m]	External bending moment
Force	$P_{y}^{*}$	[N]	First buckling load

## Data Availability

The original contributions presented in this study are included in the article. Further inquiries can be directed to the corresponding author.

## Appendix A

In order to use Equation (62) with data deriving from experimental measures, the propagation of errors was calculated here in Appendix A. More specifically, it was assumed that the following data were obtained from experiments:

(A1) $$\begin{array}{c} E=\bar{E}\pm \sigma_{E}\\ R=\bar{R}\pm \sigma_{R}\\ L=\bar{L}\pm \sigma_{L} \end{array}$$

where $\bar{E}$, $\bar{R}$, and $\bar{L}$ were the mean values of stiffness, radius and total length of the spine, while $\sigma_{E}$, $\sigma_{R}$, and $\sigma_{L}$ were their standard deviations. In this case, Equation (62) was rewritten as the following:

(A2) $$P_{y}^{*}=\bar{P_{y}^{*}}+\Delta P_{y}^{*}$$

where:

(A3) $$\bar{P_{y}^{*}}=N^{2}\frac{\bar{E}{\bar{R}}^{4}}{{\bar{L}}^{2}}$$

is the mean value, while

(A4) $$\Delta P_{y}^{*}=N^{2}\frac{\bar{E}{\bar{R}}^{4}}{{\bar{L}}^{2}}\sqrt{{\left[\frac{\sigma_{E}}{\bar{E}}\right]}^{2}+{\left[\frac{4\sigma_{R}}{\bar{R}}\right]}^{2}+{\left[\frac{2\sigma_{L}}{\bar{L}}\right]}^{2}}$$

is the resulting standard deviation. For the following values *E* = 17.40 ± 3.60 GPa [93], *R* = 0.30 ± 0.03 mm, and *L* =8.46 ± 1.56 mm [93], Equation (A2) results in:

(A5) $$P_{y}^{*}=N^{2}\left(1.97\pm 0.60\right)$$

where $N^{2}$ is evaluated through Equation (62) together with one of the following Equations (12), (28), (41) and (60), to be selected according to the correct boundary conditions.
